# Prioritization of Free-Text Clinical Documents: A Novel Use of a Bayesian Classifier

**DOI:** 10.2196/medinform.3793

**Published:** 2015-04-10

**Authors:** Mark Singh, Akansh Murthy, Shridhar Singh

**Affiliations:** 1 Carnegie Mellon University University of Massachusetts Medical School Braintree, MA United States; 2 Massachusetts Institute of Technology Cambridge, MA United States; 3 Carnegie Mellon University Pittsburgh, PA United States

**Keywords:** clinical reports, prioritization, Bayesian classifier, radiology, natural language processing

## Abstract

**Background:**

The amount of incoming data into physicians’ offices is increasing, thereby making it difficult to process information efficiently and accurately to maximize positive patient outcomes. Current manual processes of screening for individual terms within long free-text documents are tedious and error-prone. This paper explores the use of statistical methods and computer systems to assist clinical data management.

**Objective:**

The objective of this study was to verify and validate the use of a naive Bayesian classifier as a means of properly prioritizing important clinical data, specifically that of free-text radiology reports.

**Methods:**

There were one hundred reports that were first used to train the algorithm based on physicians’ categorization of clinical reports as high-priority or low-priority. Then, the algorithm was used to evaluate 354 reports. Additional beautification procedures such as section extraction, text preprocessing, and negation detection were performed.

**Results:**

The algorithm evaluated the 354 reports with discrimination between high-priority and low-priority reports, resulting in a bimodal probability distribution. In all scenarios tested, the false negative rates were below 1.1% and the recall rates ranged from 95.65% to 98.91%. In the case of 50% prior probability and 80% threshold probability, the accuracy of this Bayesian classifier was 93.50%, with a positive predictive value (precision) of 80.54%. It also showed a sensitivity (recall) of 98.91% and a F-measure of 88.78%.

**Conclusions:**

The results showed that the algorithm could be trained to detect abnormal radiology results by accurately screening clinical reports. Such a technique can potentially be used to enable automatic flagging of critical results. In addition to accuracy, the algorithm was able to minimize false negatives, which is important for clinical applications. We conclude that a Bayesian statistical classifier, by flagging reports with abnormal findings, can assist a physician in reviewing radiology reports more efficiently. This higher level of prioritization allows physicians to address important radiologic findings in a timelier manner and may also aid in minimizing errors of omission.

## Introduction

### Data Concerns

In today’s environment with electronic medical records (EMR) gaining prevalence in hospitals, urgent care clinics, and specialist facilities, primary care physicians are receiving more clinical reports on a daily basis. These electronic systems typically generate more pages per report than in the past. A Brigham and Women’s Hospital study reports that full-time primary care physicians on average review 930 pieces of chemistry/hematology data and 60 pathology/radiology reports in a given week [1]. Also, with the increasing utilization of new imaging modalities, such as computerized axial tomography (CAT) scans, magnetic resonance images (MRI), and pet scans in addition to traditional plain film studies, physicians have to process more types of reports, manage incidental findings, as well as significant findings that may require follow-up over an interval of a few weeks to even years. To compound matters, there are also more numbers of insured patients coming into the medical care community [2]. Given the existing data load and a potential increase in data [3], it will be challenging for a physician to keep up with the workload efficiently. Consequently, it is not uncommon even now for a clinician to overlook or fail to address an abnormal result. In the outpatient settings, between 8% and 26% of abnormal test results, including those suspicious for malignancy, are not followed up in a timely manner [4]. Failing to do so can result in patient morbidity and mortality, as well as possible costly malpractice litigation.

In fact, failure to review and follow up on an outpatient test result compromises patient safety and raises malpractice concerns in the order of billions of dollars annually [5]. A regional Veterans Administration health care network study indicated that almost 65% of diagnostic errors are due to abnormal test results that were missed and not addressed appropriately [6,7]. Despite the greater availability of EMR with test result transmission and notification availability, the problem of missed test results has not been eliminated. This missing of abnormal results was true even when one or more providers read the results. Alert fatigue, an inevitable presence with multiple electronic systems, is also a huge concern, especially since it results in physicians ignoring vital alerts about patients [8]. Overlooking these key recommendations or findings contained in a report, such as detection of an early cancer or a new medical condition results in adverse patient outcomes, annually, more than 100,000 patient deaths [5]. Thus, there exists a critical need for a more reliable method of clinical report management.

### Literature Review

Currently, patient clinical data are both structured and unstructured. Structured patient data are typically a laboratory test containing discrete numerical values. An example can be a patient’s potassium result, which could have a value of “4.2”. Discrete results can easily be identified and traced by automatic systems. Urgent or critical laboratory values can be detected by performing a simple numerical comparison. A problem exists, however, with free-text reports such as radiology results. These reports have to be read by the physician and important findings need to be noted and logged for proper tracking [9]. Automatic interpretation of these free-text reports and determination of whether they contain a critical finding has been challenging for computers.

There are many existing applications that use natural language processing (NLP) to extract patient medical information from free-text reports for purposes of medical billing or populating a patient's health record. Several studies have also demonstrated the ability of NLP to extract clinical information, such as pneumonia cases, from radiology reports [10,11]. Another study validated the use of a Bayesian classifier to identify the diagnosis of appendicitis from radiology reports based on training data [12]. Further experiments have demonstrated the feasibility of using statistical text classification to detect severe extreme-risk events in clinical incident reports [13,14]. However, current literature does not contain within it an application that classifies a real-time stream of incoming free-text radiology reports, automatically flags critical reports as high-priority, and learns from the physician’s actions.

By performing an initial screen of incoming data and flagging reports as potentially low-priority or high-priority, a classifier can aid physicians in better prioritization of his or her stack of clinical data that is to be reviewed. The intent is not to replace the manual review and signing off of each report, but rather to assist the physician by providing a level of prioritization to the stack of unordered documents awaiting review, an additional safety net. The benefits of such a system would be, at the very least, quicker notification of results to a patient and fewer missed or overlooked findings, resulting in better patient outcomes and possibly even less malpractice exposure.

### Naive Bayes Approach

A statistical approach using the Bayes theorem was developed to classify free clinical reports as low- or high-priority. A naive Bayesian classifier is a probabilistic classifier based on Bayes’ theorem that makes a strong independence assumption. In context, the classifier assumes that all features of a document are independent of one another. The presence or absence of one feature is assumed to have no effect on the presence or absence of any other feature. When classifying text, each feature is an individual word in the text.

A supervised learning approach is used to enable a naive Bayes classifier to differentiate a document into different categories. In the case of classifying clinical reports, the classifier categorizes reports as low- or high-priority. The classifier is trained using a corpus of documents that is already categorized. The corpus of documents is tokenized, and each word from the documents is assigned a probability of appearing in a high-priority report. Each word, or feature, is represented by “f_i_”. The probability of a report being high-priority is given by the Bayes theorem shown here in generic form,

P(H | f_i_) = [P(f_1_, f_2_,f_3_...f_i_| H) P(H)] / 

[P(H) P((f_1_, f_2_,f_3_...f_i_| H) + (1-P(H)) (1-P(f_1_, f_2_,f_3_...f_i_| H))].

The term P(H) represents the prior probability or the probability of any given document being high-priority. P(f_i_ | H) is the probability of the feature, f_i_, appearing in a document given that the document in question is high-priority. The denominator is the probability of f_i_ appearing in any given document, or P(f_i_). Thus, the equation can be simplified into,

P(H | f) = [P(f, f,f...f| H) P(H)] / 

[P(f_1_, f_2_,f_3_...f_i_)].

The experiment is based on the premise that there are patterns within clinical reports that influence a physician’s determination of the reports severity, and these patterns can be detected by a computer based on the relative presence of certain words in documents. If true, then a computer could use principles of statistics and machine learning to prioritize free-text clinical reports.

## Methods

### Study Site

Blue Hills Medical Associates is an internal medicine practice, consisting of 2 physicians and 1 nurse practitioner situated with the encatchment area of three community hospitals, each affiliated with a separate major Massachusetts health system. The practice sees over 60 patients daily and receives over 5000 pages of clinical reports each month in the form of faxes, paper mail, and electronic results via a health level-7 (HL7) interface. These reports include consult reports, laboratory results, hospital admission and discharge reports, as well as radiology reports. The focus of this study was on the management of radiology results. There were 2 primary care physicians who reviewed the reports.

### Datasets

There were two sets of data that were used in this study. Both sets of data were extracted from clinical reports stored in the EMR at the practice site used for this study. The first set, the training data, was used to train the Bayesian classifier to detect physicians’ definitions of what constitutes a high-priority report. The second set of data, the test data, was a set of documents independent from the training set used to test and validate the classifier against the physicians’ own categorization.

Radiology reports usually have an Impression section, summarizing the report’s key findings. The Impression section is the interpreting radiologist’s summarization and prioritization of the report’s key findings. Focusing on this section allows for easier data processing, since the reports have been preprioritized by level of importance by the radiologist. The Impression section was extracted from each report for inclusion in the corpora based on the assumption that it contained the key information that distinguishes a low-priority report from a high-priority report. In the few cases where a report does not contain an Impression section or its equivalent, the entire report body was processed. The described extraction limits the amount of extraneous data.

### Training Data

**Figure 1:**
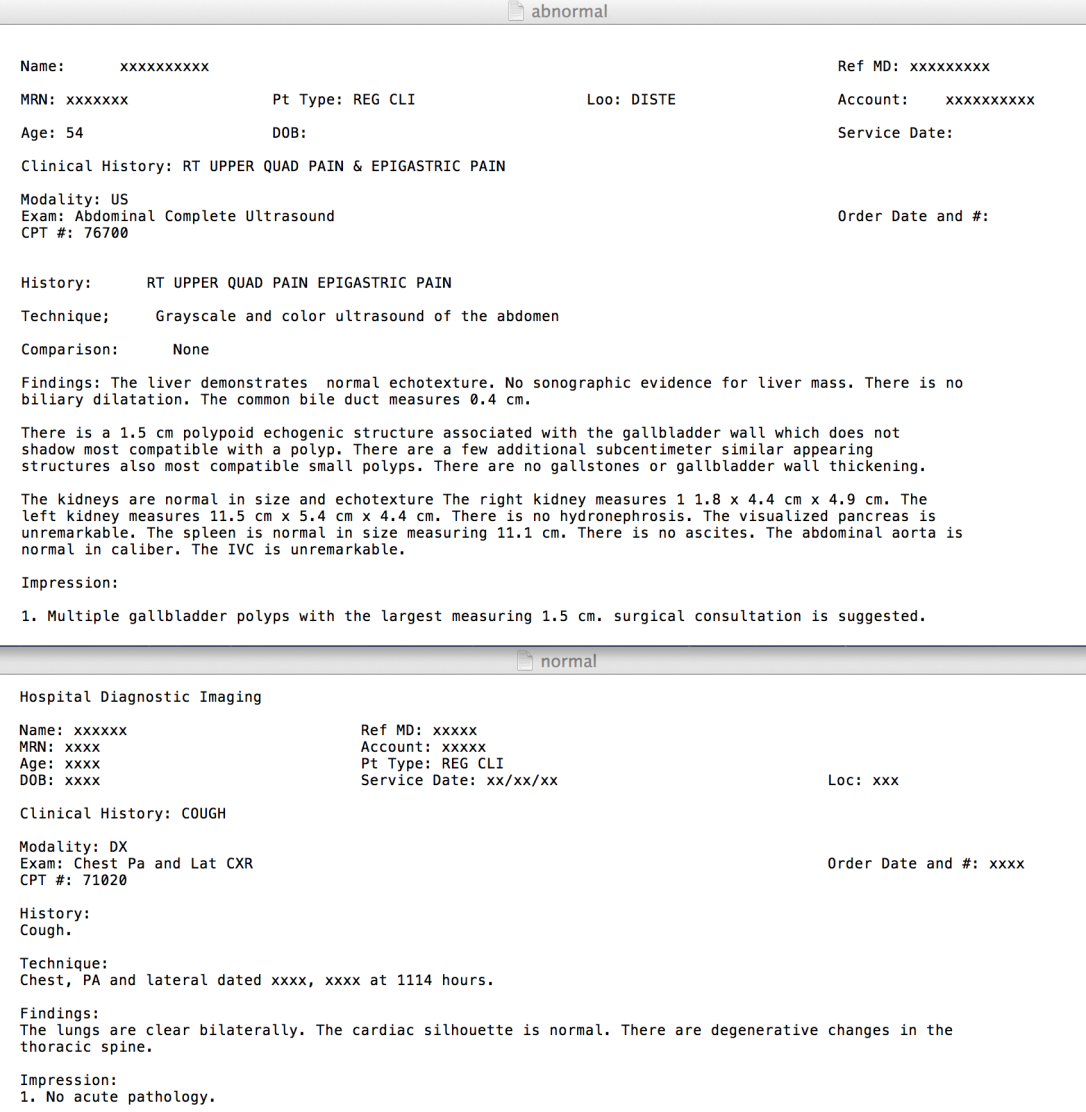
Deidentified high-priority (top) and low-priority (bottom) patient reports in text format.

There were one hundred reports, 50 from each category generated between the years of 2011 and 2013, that were selected from the EMR that were representative of the types of low- and high-priority reports seen in study site. These were then categorized into low- and high-priority by the physicians. Figure 1 shows examples of deidentified high-priority and low-priority reports in common text format.

### Test Data

There were three hundred and fifty four radiology and diagnostic reports, ordered by the practice and generated between the years 2011 and 2013, that were selected randomly out of 4800 reports to test the classifier trained by the training dataset. These reports include CAT scans of the head abdomen, MRIs of the head and neck cervical spine lumbar spine abdomen, and plain X-ray films of the chest abdomen and various extremities (Table 1)**.** They were not limited by a particular specialty, since a primary care practice patient panel is broad based and not limited by specialty.

**Table 1:** Distribution of the types of reports used in the test dataset.

Type of report	Percentage, n (%)
Mammograms	35/354 (9.9)
CAT scans	36/354 (10.2)
Plain radiology films	71/354 (20.1)
Ultrasounds	70/354 (19.8)
MRIs	142/354 (40.1)

### High-Priority Reports

Reports flagged as high-priority are those reports that require further follow-up by the primary care physician. An example is a finding of a renal cyst, which may require a 6-month follow-up ultrasound. Another example is a lung nodule, which may require a 4-month follow-up CAT scan.

### Processing Steps

**Figure 2:**
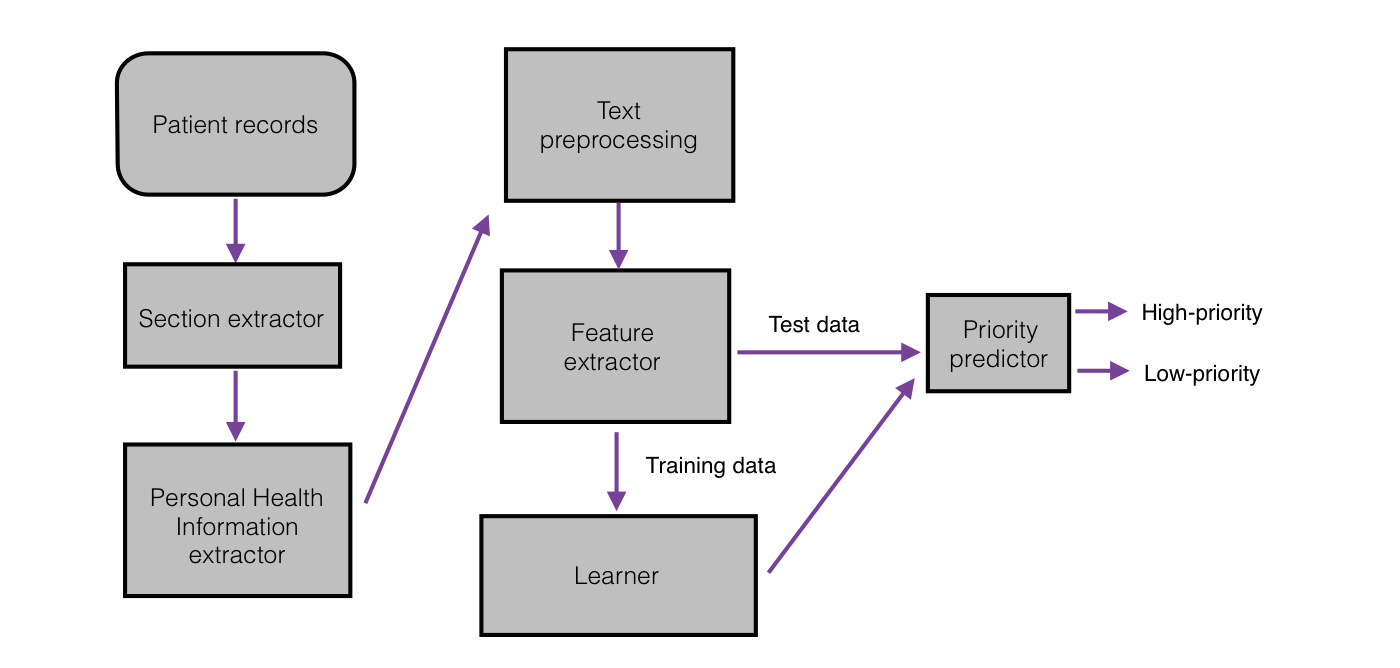
System architecture.

Figure 2 shows the main components of the system. First, the document was retrieved from the EMR, and the Impression section was extracted. The resulting data were then processed to remove any protected health information (PHI). By extracting just the Impression section of the report, much of the PHI was automatically excluded. However, in some cases, there was remaining PHI, such as patient identifying information or the name of the health care facility. Using lexical look-up tables, regular expressions, and simple heuristics described in [9], any remaining PHI was removed.

The next step was text processing and feature extraction, which began with cleanup routines such as conversion of all characters to lowercase type and the removal of stop words. Stop words are words that do not have any value in determining the priority level of a document. Examples of stop words include “the”, “it”, “of”, and “a”. Removing stop words in the preprocessing step is a common practice in artificial intelligence. Doing so minimizes the overall processing load and memory requirements, and results in a narrow set of clinically relevant terms [15]. Then, terms that were negated by negation terms were removed. Negation terms have a large effect on the meaning of sentences. For example, a high-priority report may contain the phrase “acute lung disease”, and a low-priority report may contain the phrase “no acute lung disease”. A naive Bayes classifier cannot differentiate between such distinctions, making the difference between these low-priority and high-priority reports ambiguous.

In addition, clinical reports often contain common phrases such as “otherwise normal chest” that can distinguish a high-priority report from a low-priority report. A naive Bayes classifier only extracts individual words from documents and assumes that the probability of each word being in different document categories is independent of the probabilities of other words. However, if a document contains a phrase such as “otherwise normal chest”, the individual probabilities assigned to each word in the phrase are clinically dependent on each other. Thus, common phrases were identified, and white spaces contained within these phrases in clinical reports were removed to create a single term that the classifier could recognize. A list of common phrases used in this study is provided in Table 2 below. An example of white space removal is shown below in Table 3.

**Table 2:** Examples of common phrases used in the data cleaning process.

Common phrases
“No significant abnormality is identified”
“No mammographic change or evidence of malignancy”
“No acute cardiopulmonary process”
“No acute pulmonary process”
“Within normal limits”
“Normal abdominal ultrasound”
“No acute intracranial process”
“Appropriate for age”
“Routine annual screening mammogram”
“No acute pathology”
“Correlation recommended”
“Biopsy should be performed”
“Surgical consultation is suggested”
“Appear significantly changed”

**Table 3:** Common phrase and white space removal depiction.

Common phrase before white space removal	Common phrase after white space removal
“within normal limits”	“withinnormallimits”
“Normal abdominal ultrasound”	“Normalabdominalultrasound”

### “Bag of Words”

The remaining words were stored as a “bag of words”, which is a representation of text as an unordered collection of terms that disregards word order or grammar. The naive Bayesian classifier treats each term in this “bag of words” independently from the others. The average number of total unique words in the “bag of words” per report was 684.

### P_p_ and P_th_

For the implementation of the naive Bayesian classifier, an open source, C# implementation of a spam filter algorithm [16] was repurposed. A spam filter was used as the initial code base because it is essentially a Bayesian classifier that is trained to detect text messages that a user considers to be spam based on training data. After the Bayesian filter was trained on clinical report training dataset, it was tested on the clinical report test dataset. The P_p_ and P_th_ values from the Bayesian equation were used as parameters in this study to facilitate the calculation of the precision, recall, F-measure, and accuracy values. P_p_ is the prior probability distribution as defined in the Bayesian equation [17,18]. It represents the probability that a received report is important based on past experience. Similarly, it can be thought of as a percentage that represents the level of suspicion that a document is important. This value, which can be set by the user, affects the misclassification rate of a report, because increasing its value will increase the likelihood that a report will be classified as important. To minimize the false negative rate, the prior probability should be set at a higher value, thus biasing the classifier toward classifying a given report as a positive one. P_th_ is the threshold probability distribution as defined in the Bayesian equation. It represents the probability cutoff where a document is classified as high-priority. Since a high P_th_ would result in higher false negatives, the manipulation of that parameter in this study was important. The cost of a misclassified important report, or a false negative, is much greater than a misclassification of a routine report. P_th_ also indicates the minimal probability at which a report is classified as important. The user can set this threshold value, typically to levels greater than 50%. For the purposes of this study, the value was set to a level that minimized false negatives, while keeping the false positives at a tolerable level, thereby not missing important reports, but also not contributing to alarm fatigue. P_p_ and P_th_ were used because their individual effects, when properly adjusted, could be used to compare sensitivity, and consequently, performance, of the classifier.

### Precision, Recall, F-Measure, and Accuracy

The performance of this Bayesian classifier implementation was evaluated using precision, recall, F-measure, and accuracy, standard performance measures for classification and machine learning tasks [19]. Precision is the ratio of true positives to the total number of documents classified as positives,

Precision = TP/(TP+FP),

where TP is true positive and FP is false positive.

Recall is the proportion of actual positives that are correctly identified as such,

Recall = TP/(TP+FN),

where FN is false negative.

F-Measure is the harmonic mean of the precision and recall measures,

F-Measure = (2)(precision)(recall/(precision+recall)).

Accuracy is the percentage of true positives and true negatives to the total number of reports processed.

Accuracy = TP+TN/(TP+TN+FP+FN).

## Results

### Randomly Selected Reports

In this study, 354 radiology reports, randomly selected from the date range of 2011 to 2013, were tested to evaluate the performance of the Bayesian classifier in detecting high-priority reports. The classifier was trained on data, preclassified by physicians, whose interrater reliability was a Cohen’s kappa value of 0.86. This training set consisted of 50 low-priority and 50 high-priority radiology reports randomly selected from the same time range. The performance of the algorithm was tested under 2 independent conditions, the prior probability of a report being high-probability or P_p,_ and the probability threshold P_th_, at which a report is classified as high-probability. Tests were run for 2 possible values for each of these variables, giving a total of 4 sets of results for analysis.

### P_p_ and P_th_

**Figure 3:**
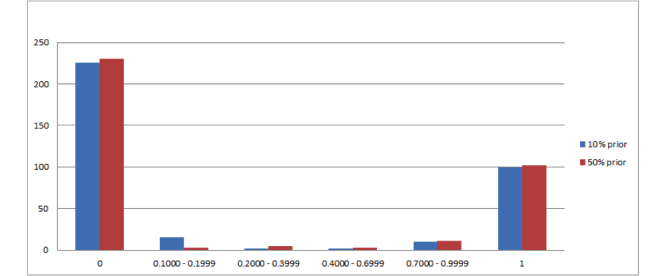
Distribution of reports in each probability range. The x-axis represents probability and the y-axis represents number of reports from the test set.

The probability of each report being high-priority was determined using Bayes formula as described in the Introduction. The distribution of the probabilities of each report being high-priority is shown below for each of the prior probabilities (P_p_) (Figure 3 shows this).

The frequency distribution of the radiology reports for each calculated probability range shows a clustering of reports at both extremes, with the majority of reports having a probability of 0 or 1, and the fewest number of reports being in the mid probability ranges from 0.2000 to 0.6999.

### Precision, Recall, F-Measure, and Accuracy Values for P_p_=10%

Table 4 lists the classifier metrics for prior probability of 10%, of being a document classified as high-priority, and for each cutoff threshold probability of 50% and 80%.

**Table 4:** Precision, recall, F-measure, and accuracy values for the classifier with P_p_= 10%.

	50% P_th_	80% P_th_
10% P_p_ of high-priority report	TP 89, FP 22, TN 240, FN 3	TP 88, FP 21, TN 241, FN4
Precision, %	80.18	80.73
Recall, %	96.74	95.65
F-measure, %	87.66	87.56
Accuracy, %	92.94	92.94

### Precision, Recall, F-Measure, and Accuracy Values for P_p_=50%

As can be seen, the precision, recall, F-measure, and accuracy values were similar for either probability thresholds given that the P_p_ was 10%. The accuracy rates for both thresholds are above 90%, but in both situations, there are a few false negatives. Table 5 lists these measures for prior probability of 50%, of being a document being classified as high-priority, and for each cutoff threshold of 50% and 80%.

In the situation of P_p_ being 50%, the precision, recall, F-measure, and accuracy values are noticeably different. The number of false negatives was the lowest at P_p_ of 50% for both values of P_th._ In fact, there was only 1 false negative in each threshold probability scenario resulting in a 0.28% false negative rate. This is also reflected in the recall rate, which decreases as the number of false negatives increases, as seen when comparing Tables 4 and 5.

**Table 5:** Precision, recall, F-measure, and accuracy values for the classifier with P_p_= 50%.

	50% P_th_	80% P_th_
50% P_p_ of report being high-priority	TP 91, FP 25,TN 237, FN 1	TP 91, FP 22, TN 240, FN 1
Precision, %	78.45	80.53
Recall, %	98.91	98.91
F-measure, %	87.50	88.78
Accuracy, %	92.66	93.50

## Discussion

### Principal Results

The results indicate that a naive Bayesian classifier works remarkably well in classifying radiology reports as low-priority or high-priority. The recall rate varied from 95.65% to 98.91%. This signifies that the classifier succeeded in accurately detecting high-priority reports, also known as true positives, while minimizing false negatives. The rate of false negatives was very low in this study, with the number of false negatives varying from 1 to 4. As indicated earlier, a lower false negative rate is desirable in clinical contexts and the current application. The precision rate, however, was lower, varying from 78.45% to 80.73%. In other words, the classifier had a higher rate of false positives. That is, it classified a greater number of documents as being high-priority, even though they were actually low-priority.

The actual magnitude of change in performance was not too dramatic for the 2 values of P_th_ (50% and 80%). The reason for this is seen by observing the nearly bimodal distribution of reports (Figure 2) falling under the extremes at probabilities of 0 and 1, with few in between.

Similar observations can be made about varying the P_p_. This is the prior probability used in the Bayes formula to calculate the probability that a report is high-priority. Increasing this value increases the likelihood that a report is high-priority. Choosing a higher P_p_ will have the effect of potentially increasing the false positive rate in the same way as it did when the threshold, P_th_, was increased. The data also demonstrated this, but again, modestly. The bimodal distribution described earlier again shows why this was the case.

This study showed a clear distinction between low-priority and high-priority reports. Why was there such a clear distinction? Low-priority reports are the normal reports. They typically have text in the Impression section such as “no evidence of fracture” or “no acute disease of the chest”. Our negation algorithm removed all negated terms. So these normal reports were presented as empty text to the Bayesian classifier. The text from a high-priority report would typically have language such as “nodule identified”, “possible developing mass”, or “small infiltrate suggesting early pneumonia”. Since these terms are not seen in a low-priority report, the classifier assigns a very high probability to reports containing these terms. Furthermore, by removing negated terms, we greatly improved the scores of the training dataset. Removing stop words had minimal impact on the document scores, but it rendered a cleaner “bag of words” to study and debug.

A closer review of the reports indicates the reason for a higher number of false positives. A false positive report for a mammogram read,

...there is no mammographic evidence of malignancy; routine follow-up mammogram in 1 year is recommended; bi-rads category 1. negative according to the nci model the patients lifetime risk of developing breast cancer is 3.6%...

Patient laboratory report

Although this report should have been classified as low-priority, there was language used by the radiologist to provide general guidance to the ordering physician, *...according to the nci model the patients lifetime risk of developing breast cancer is 3.6%*. The classifier identified the term, “cancer”, and assigned a high probability to the report. In another case, a report read, “chest is without evidence of pneumonia”. Our classifier did not properly detect the negation term “without”, and thus the term “pneumonia” resulted in a false positive.

### Limitations

More robust negation detection should be developed as a part of any future enhancements. Additionally, use of NLP and/or common phrase detection may enhance the ability of the classifier to better distinguish if terms mentioned are part of a patient’s report findings, as in the case described above. Additional statistical methods, in addition to Bayesian statistics, could also provide a stronger classification system.

Although this study had a relatively low number of documents to test and a small number of reviewers, the superior results obtained provide assurance for the performing of future studies and make intuitive sense for the nature of the primary care setting. Patients in this setting are generally healthy and tend to have normal radiology reports. The distinction between a normal and an abnormal report is usually obvious due to the presence of key words, making it easy for a classifier to detect an abnormal report and denote it as high-priority.

### Differences From Prior Work

The results of this study highlight the promise of using statistical classifiers, such as this Bayesian implementation, in prioritizing a primary care physician’s workload across electronic systems in real-time with an ability to be trained, a marked difference from the retrospective and static analyses done by many of the prior studies in the literature. Due to the EMR-agnostic design of this classifier, it is generalizable to any EMR system or patient data interface for that matter. The real-time incoming data feed to an EMR can consist of various entry points, such as HL7, faxes, scanned documents, and Web services. This classifier might also offset the chance of radiologists not electronically coding radiology reports as normal or abnormal, as these specialists are typically required by the American College of Radiology to electronically code radiology reports as normal or abnormal when communicating with primary care physicians [20]. Even in the case of proper coding, this classifier can act as an additional layer of safety and clinical intelligence with minimal infrastructure and integration costs that are typical of many of the reviewed software systems of the past. Ultimately, use of this tool for prioritizing the physician’s workload and aiding in the detection of abnormal radiologic, as well as other findings, can greatly enhance patient safety.

While the scope of documents was limited in this study to radiology, we believe the classifier can be adapted to other verticals within health care. Implementing it on a greater number of radiology reports and testing it on other report types, such as pathology and microbiology reports, will further test the effectiveness of the Bayesian classifier in this study.

### Conclusions

In conclusion, a Bayesian classifier can be used, in conjunction with other available methods, to detect high-priority radiology reports and improve primary care provider efficiency in addressing these reports. This novel study showed, for the first time to our knowledge, that the Bayesian system, used on this representative sample of free text, unstructured radiology reports received in a primary care setting, displayed a high rate of success in detecting true positives. Use of this type of technology has the potential to improve patient safety, as well as minimize physician malpractice exposure.

Future work may include studying the effectiveness of this classifier in a different practice setting, such as a specialist’s office. For example, in an oncology or cardiology practice, given the nature of each specialty, a greater number of patient reports are expected to be abnormal, and yet may be classified by the specialist as low-priority. It would be interesting to see how this classifier would perform. It is possible that more advanced techniques such as NLP, in combination with a statistical classifier, would be required in order to have a satisfactory rate of high-priority detection. Furthermore, search engine capabilities could be a future extension as specific terms within reports can be identified, leading to a more connected experience for the patient [21]. Such an application might be able to assist in recording and analysis of a long-term view of high-priority events or even disease maps based on the terms that have been flagged, resulting in better visualizations for value-based care or pharmaceutical drug targeting. More immediately, this study makes clear that the intersection of computer science, statistics, and health care can have huge implications that can improve efficiency, patient safety, and quality of care.

## Abbreviations

CAT: computerized axial tomography
EMR: electronic medical records
HL7: health level-7
MRI: magnetic resonance images
NLP: natural language processing
PHI: protected health information
