# Correction: Prioritization of Free-Text Clinical Documents: A Novel Use of a Bayesian Classifier

**DOI:** 10.2196/21379

**Published:** 2020-06-23

**Authors:** Mark Singh, Akansh Murthy, Shridhar Singh

**Affiliations:** 1 Carnegie Mellon University University of Massachusetts Medical School Braintree, MA United States; 2 Massachusetts Institute of Technology Cambridge, MA United States; 3 Carnegie Mellon University Pittsburgh, PA United States

In “Prioritization of Free-Text Clinical Documents: A Novel Use of a Bayesian Classifier” (JMIR Med Inform 2015;3(2):e17) the authors noted an error in corresponding author’s details.

The corrected information is:

Akansh Murthy, BS

Massachusetts Institute of Technology

77 Mass Ave

Cambridge, MA, 02139

United States

Phone: 1 6172531000

Email: ambshun@mit.edu

The correction will appear in the online version of the paper on the JMIR Publications website on June 23, 2020, together with the publication of this correction notice. Because this was made after submission to PubMed, PubMed Central, and other full-text repositories, the corrected article has also been resubmitted to those repositories

